# Effects of Passage Number and Differentiation Protocol on the Generation of Dopaminergic Neurons from Rat Bone Marrow-Derived Mesenchymal Stem Cells

**DOI:** 10.3390/ijms19030720

**Published:** 2018-03-02

**Authors:** Gabrielle Shall, Megan Menosky, Sarah Decker, Priya Nethala, Ryan Welchko, Xavier Leveque, Ming Lu, Michael Sandstrom, Ute Hochgeschwender, Julien Rossignol, Gary Dunbar

**Affiliations:** 1 Field Neurosciences Institute Laboratory for Restorative Neuroscience, Central Michigan University, Mount Pleasant, MI 48859, USA; shall1gp@cmich.edu (G.S.); menos1ml@cmich.edu (M.M.); decke1sn@cmich.edu (S.D.); pnethala@gmail.com (P.N.); welch1rm@cmich.edu (R.W.); xavierleveque2@gmail.com (X.L.); rossi1j@cmich.edu (J.R.); 2 Neuroscience Program, Central Michigan University, Mount Pleasant, MI 48859, USA; lu1m@cmich.edu (M.L.); sands1m@cmich.edu (M.S.); hochg1u@cmich.edu (U.H.); 3 College of Humanities and Social and Behavioral Sciences, Psychology Department, Central Michigan University, Mount Pleasant, MI 48859, USA; 4 College of Medicine, Central Michigan University, Mount Pleasant, MI 48859 USA; 5 Field Neurosciences Institute, 4677 Towne Centre Rd. Suite 101, Saginaw, MI 48604, USA

**Keywords:** Parkinson’s disease, mesenchymal stem cells, dopaminergic neurons, passaging, induction protocol

## Abstract

Multiple studies have demonstrated the ability of mesenchymal stem cells (MSCs) to differentiate into dopamine-producing cells, in vitro and in vivo, indicating their potential to be used in the treatment of Parkinson’s disease (PD). However, there are discrepancies among studies regarding the optimal time (i.e., passage number) and method for dopaminergic induction, in vitro. In the current study, we compared the ability of early (P4) and later (P40) passaged bone marrow-derived MSCs to differentiate into dopaminergic neurons using two growth-factor-based approaches. A direct dopaminergic induction (DDI) was used to directly convert MSCs into dopaminergic neurons, and an indirect dopaminergic induction (IDI) was used to direct MSCs toward a neuronal lineage prior to terminal dopaminergic differentiation. Results indicate that both early and later passaged MSCs exhibited positive expression of neuronal and dopaminergic markers following either the DDI or IDI protocols. Additionally, both early and later passaged MSCs released dopamine and exhibited spontaneous neuronal activity following either the DDI or IDI. Still, P4 MSCs exhibited significantly higher spiking and bursting frequencies as compared to P40 MSCs. Findings from this study provide evidence that early passaged MSCs, which have undergone the DDI, are more efficient at generating dopaminergic-like cells in vitro, as compared to later passaged MSCs or MSCs that have undergone the IDI.

## 1. Introduction

Parkinson’s disease (PD) is the second most common neurodegenerative disorder after Alzheimer’s disease, with the incidence of PD rapidly increasing in individuals over the age of 60 [1,2]. The pathological hallmark of PD is the loss of dopaminergic neurons from the substantia nigra, pars compacta that innervate the striatum through the nigrostriatal pathway [3]. This degeneration is often accompanied by an intraneuronal accumulation of α-synuclein-containing Lewy bodies in the remaining, intact nigral neurons [2,3,4,5]. The characteristic deafferentiation of the basal ganglia leads to the recognizable motor-related symptoms of PD, including resting tremor, muscular rigidity, postural instability, and bradykinesia [6,7,8,9,10,11]. Although the causes of PD have not been completely elucidated, predispositions for this disease are thought to be both environmental and genetic [12,13].

Due to the extensive degeneration of midbrain nigral neurons in PD, cell-based therapies have been gaining more attention as a way of replacing these lost neurons and restoring motor-function in patients suffering from this disease. To date, studies have provided evidence that dopaminergic neurons, derived from the human fetal mesenchephalon, are able to reinnervate the damaged striatum and ameliorate the symptoms of PD [14,15,16,17,18,19,20,21]. More recently, researchers have indicated the ability of induced pluripotent stem cells (iPSCs) to differentiate into dopaminergic neurons [22,23,24]. Still, there are ethical concerns, the risk of graft-induced dyskinesias (GID), and issues of tissue rejection that are associated with the utilization of the human embryonic brain as a source of stem cells [20,25,26,27]. Additionally, transplantations of iPSCs introduce the risk of tumor formation in vivo [28,29,30,31,32,33]. These issues warrant the use of alternative sources for cell replacement therapies.

Mesenchymal stem cells (MSCs) have been identified as a potential candidate for use in regenerative medicine. MSCs can be isolated from a variety of sources, are easily expandable in culture, and exhibit immunomodulatory properties in vivo, making them ideal for cell transplantation studies [13,34,35,36,37,38]. Additionally, MSCs have been shown to aid in neural repair through the secretion of cytokines and neurotrophic factors [35,39,40,41] and may also hold the potential to transdifferentiate into cells of ectodermal origin, such as neural stem cells, neurons [42,43,44,45,46,47,48,49,50,51,52,53], and more specifically, dopaminergic neurons [5,10,54,55,56,57,58,59,60,61]. Still, there are several concerns surrounding this area of research, including the optimal method for generating dopaminergic neurons from MSCs and the optimal time, in terms of passage number, to initiate neuronal differentiation of MSCs. 

Emerging research is suggesting that passage number may significantly influence the neuronal differentiation capacity of MSCs. Because there is a low frequency of MSCs in bone marrow, it is often necessary to expand these cells, in vitro, prior to clinical use or transplantations in animal models [62,63,64]. Interestingly, many studies have indicated that long-term serial passaging (*p* > 8) in vitro may attenuate the expression of cytokines and specific surface markers, induce rapid aging and accelerated senescence, and affect the morphology and proliferative capacity of MSCs [62,63,64,65,66,67,68,69]. Additionally, multiple studies have reported that the efficiency of neural transformation of MSCs is decreased with increased expansions [62,65,70]. Conversely, others have suggested that extended subculturing of these cells does not compromise their ability to develop toward an early neuronal fate [69,71]. For instance, Khoo and colleagues [64] determined that long-term serial passaging of MSCs resulted in changes in proliferative ability and symmetric division, but these cells were still able to undergo morphological and gene expression changes toward neuronal-like cells, similar to early passaged cultures. In contrast, Zhang and colleagues [70] demonstrated that neural-like cells could be generated from early-passaged (*p* < 8) human MSCs, but the capacity for neural differentiation declined with passaging. Researchers suggest that the higher plasticity of early-passaged MSCs may be explained by the low to moderate expression of several pluripotent and neural genes that are diminished at late passages [63,64,70].

Another issue regarding neuronal conversion of MSCs is the ability of MSCs to differentiate into dopaminergic neurons. Although there are increasing reports that claim the generation of dopaminergic neurons from MSCs, there are still discrepancies regarding the optimal method for developing dopaminergic (DA) neurons from these cells. For example, Trzaska and colleagues [57,60] demonstrated that dopaminergic neurons could be directly generated from human bone marrow-derived MSCs (i.e., percentage of TH-expressing cells) after being treated with growth factors involved in dopaminergic production during development, such as sonic hedgehog (SHH) and fibroblast growth factor-8 (FGF-8). Their results indicated that the differentiated MSCs expressed significantly higher levels of tyrosine hydroxylase (TH), dopamine transporter (DAT), LIM homeobox transcription factor 1 (Lmx1a), nuclear receptor related-1 protein (Nurr1), and pituitary homeobox 3 (Pitx3) mRNA compared to untreated MSCs, and were also shown to secrete DA [57,60]. Additionally, Fu and Colleagues [61] were able to directly induce rat bone marrow- derived MSCs into dopaminergic neurons following exposure to growth factors for 12 days. Alternatively, some researchers have indicated that it may be necessary to first direct MSCs toward a neural stem cell-like population lineage prior to terminal dopaminergic differentiation [5,54,55,56,58].

A number of studies have implemented multiple stage induction protocols, whereby cells are first exposed to growth factors that initiate neural stem cell/neurosphere formation and are then subsequently treated with factors involved in the production of dopaminergic neurons [5,58]. Specifically, Fu and colleagues [55] developed a three-stage method for the generation of dopaminergic neurons from human umbilical cord mesenchymal stem cells (hUMSCs). Similarly, following a multiple-stage induction, Khoo and colleagues [5] demonstrated that neurosphere-like clusters were formed following exposure of human bone marrow MSCs to growth factors involved in neural stem cell production, but MSCs lacked the positive expression of mature dopaminergic markers following treatment with SHH and FGF-8.

**Figure 1:**
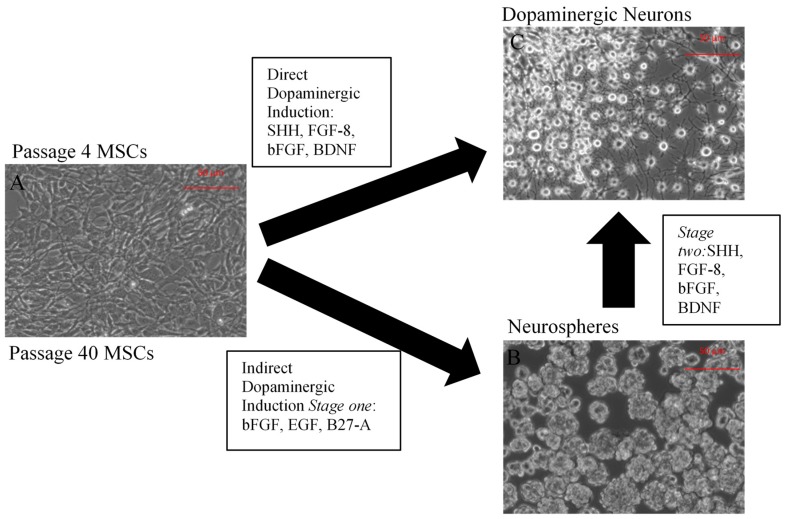
Overview of the direct dopaminergic induction (DDI) and indirect dopaminergic induction (IDI). Undifferentiated P4 or P40 BM-MSCs (**A**). Neurosphere formation following Stage One of the IDI (**B**). Dopaminergic neurons following the DDI or Stage Two of the IDI (**C**).

The present study explored the capacity of MSCs to differentiate into a neuronal phenotype and compared the efficiency of early (P4) and later (P40) passaged MSCs to differentiate into dopaminergic neurons when using a direct (DDI) or an indirect dopaminergic induction (IDI) method (Figure 1). Our results demonstrate that early and later passaged MSCs express neuronal and dopaminergic-specific genes, secrete dopamine, and exhibit electrophysiological activity following either the DDI or IDI. However, passage number appears to influence electrophysiological properties of induced MSCs.

## 2. Results

### 2.1. Neuronal Differentiation of Mesenchymal Stem Cells (MSCs)

#### 2.1.1. Characterization of Undifferentiated MSCs

Following isolation and expansion of MSCs, flow cytometry (FACs) analyses were performed to identify the expression of MSC cell surface markers. Results from FACs revealed that both P4 and P40 MSCs showed positive expression for the MSC marker, CD90, but low expression for the MSC marker, CD105 (Table 1). Interestingly, P4 and P40 MSCs also showed positive expression for the immature neuronal markers, TUJ1 and Nestin (Table 1). Additionally, P4 and P40 undifferentiated MSCs were negative for the hematopoietic stem cell marker, CD34, and the major histocompatibility complex class II (MHC II) antigen (Table 1), which is consistent with MSC characterization.

#### 2.1.2. Direct Dopaminergic Induction

**Figure 2:**
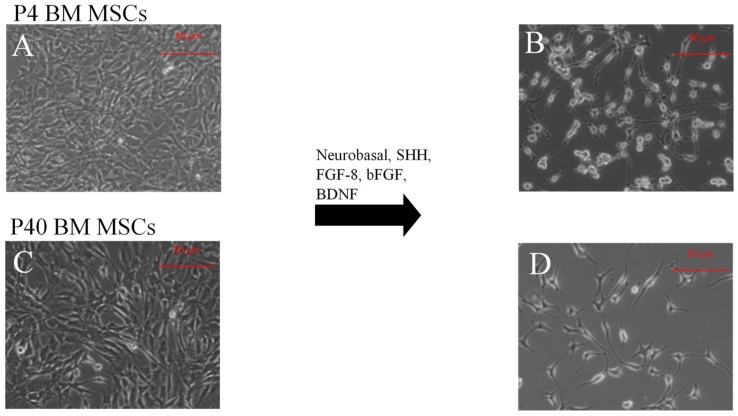
Morphological changes of MSCs following the DDI. Passage 4 (**A**) and passage 40 (**C**) BM-derived MSCs before induction. Following exposure to SHH, FGF-8, bFGF, and BDNF, both P4 and P40 MSCs exhibited changes in morphology toward a neuronal phenotype. (**B**) P4 MSCs post-differentiation, (**D**) P40 MSCs post-differentiation.

At the beginning of induction, both P4 and P40 MSCs cultures phenotypically consisted of a relatively homogeneous population of cells. Morphologically, cells exhibited large, thin flattened cell bodies, with large nuclei (Figure 2A,C). MSCs that underwent the DDI showed a change in morphology at 48–72 h following treatment with SHH, FGF-8, and basic fibroblast growth factor (bFGF). At this time, both populations of MSCs exhibited spherical refractile cell bodies, with dendritic-like processes, and long thin axonal-like projections from the cell soma, typical of neurons (Figure 2B,D). The MSCs retained this morphology for the duration of the induction.

#### 2.1.3. Indirect Dopaminergic Induction

**Figure 3:**
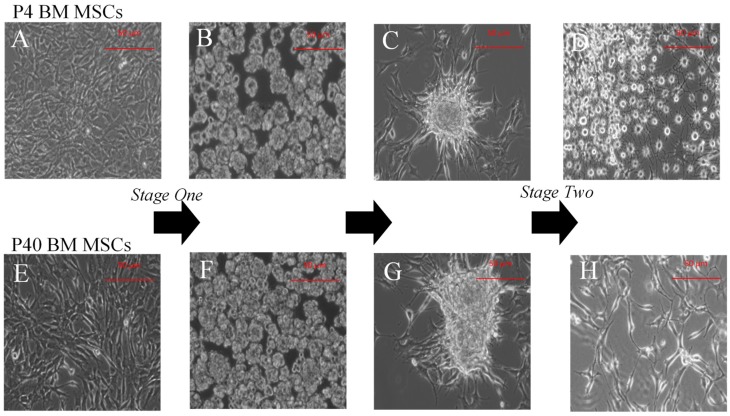
Morphological changes throughout the IDI. P4 (**A**) and P40 (**E**) MSCs before induction. Following exposure to bFGF, EGF, and B-27 (without Vitamin A), both P4 (**B**) and P40 (**F**) MSCs formed free-floating spheres. Cells adhered to poly-l-ornithine-coated coverslips and single cells grew outward from the attached sphere; ((**C**) P4 NSCs) ((**G**) P40 NSCs). Following exposure to SHH, FGF-8, bFGF, and BDNF, both P4 and P40 MSC displayed changes in morphology toward a neuronal phenotype ((**D**) P4 MSCs post-induction, (**H**) P40 MSCs post-induction).

MSCs that underwent the IDI remained detached from the culture flask and developed into free-floating clusters of cells (i.e., neurospheres) at 48 h following treatment with B27-A, epidermal growth factor (EGF), and bFGF (Figure 3B,F). For terminal dopaminergic differentiation, the neurospheres adhered to the culture flask and grew outward from the attached sphere (Figure 3C,G). Following treatment with SHH, FGF-8, and brain-derived neurotrophic factor (BDNF), cells displayed a neuronal-like morphology (Figure 3D,H).

### 2.2. MSCs Express Neuronal and Dopaminergic-Specific Markers Following DDI and IDI

#### 2.2.1. Immunophenotype of MSCs following the Direct Dopaminergic Induction

**Figure 4:**
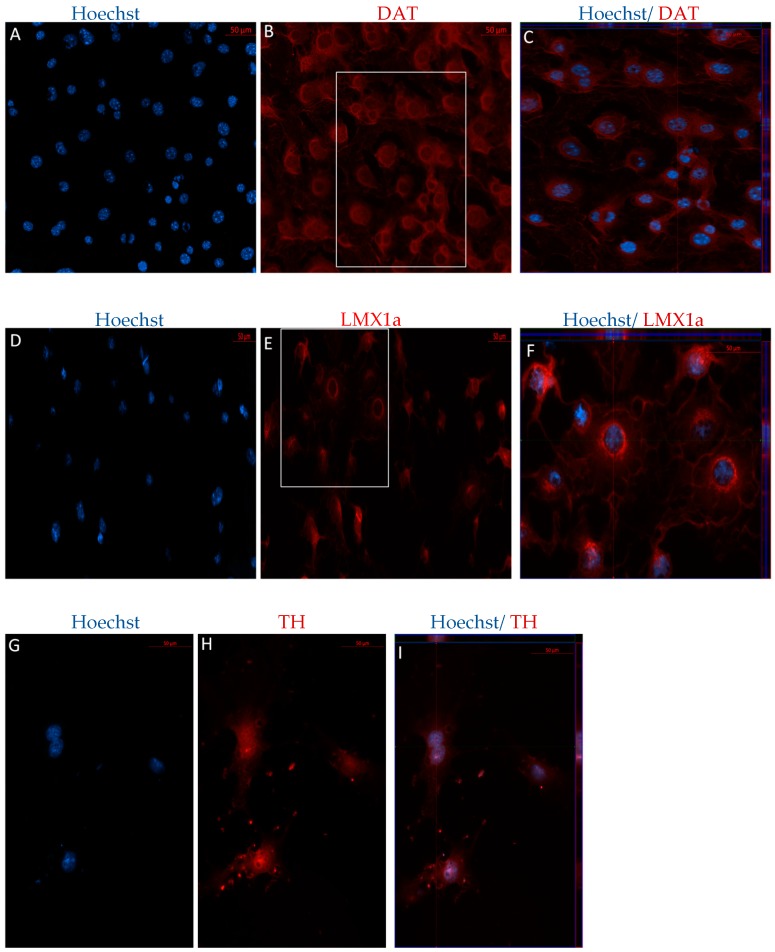
Immunocytochemistry/immunofluorescence (ICC/IF) performed on P4 MSCs following the DDI. Hoechst-labeled P4 MSCs (**A**,**D**,**G**) revealed positive staining of the mature dopaminergic markers DAT ((**B**) red) and TH ((**H**) red), and the immature dopaminergic marker LMX1a ((**E**) red), following exposure to SHH, FGF-8, bFGF, and BDNF. Orthogonal displays of *z*-stacks are represented in merged images (**C**,**F**,**I**).

**Figure 5:**
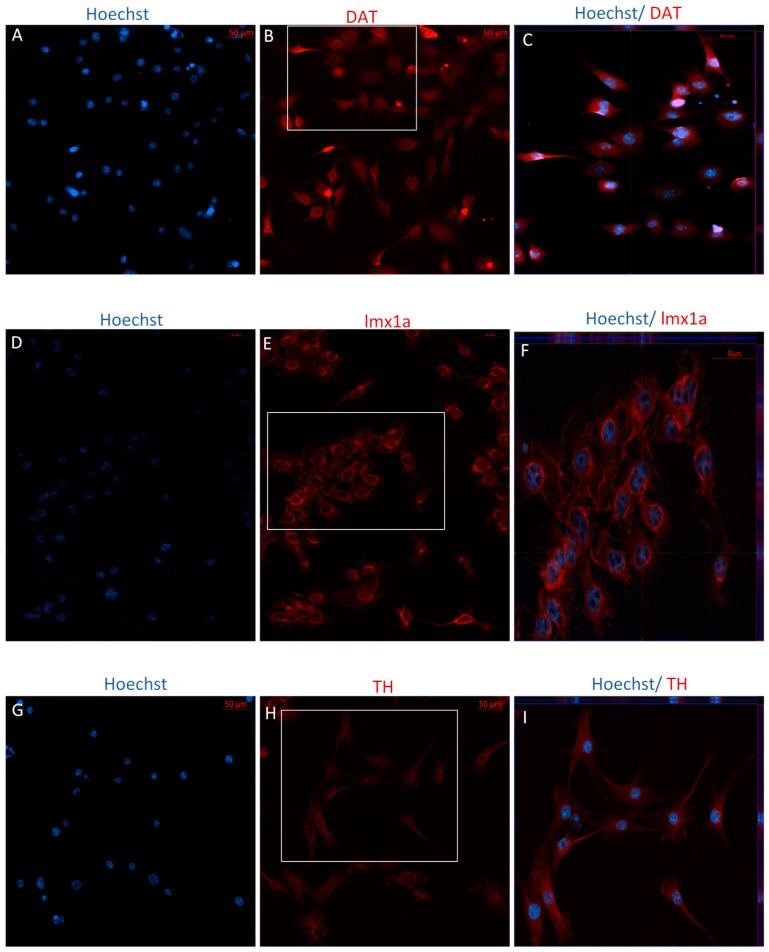
Immunocytochemistry/immunofluorescence (ICC/IF) performed on P40 MSCs following the DDI. Hoechst-labeled P40 MSCs (**A**,**D**,**G**) revealed positive staining of the mature dopaminergic markers DAT ((**B**) red) and TH ((**H**) red), and the immature dopaminergic marker LMX1a ((**E**) red), following exposure to SHH, FGF-8, bFGF, and BDNF. Orthogonal displays of *z*-stacks are represented in merged images (**C**,**F**,**I**).

Following differentiation, MSCs were characterized for the expression of dopaminergic and neuronal markers using immunocytochemistry (ICC) and flow cytometry. After 12 days in vitro, positive staining for the immature dopaminergic marker, LMX1a, and the mature dopaminergic markers, TH and DAT, was identified in both early and later passaged MSCs following the DDI (Figure 4 and Figure 5). Further, flow cytometry analyses revealed that directly differentiated P4 MSCs exhibited an increase in the expression of TH, DAT, and microtubule-associated protein 2 (MAP2) but a decrease in Nestin expression, as compared to undifferentiated control P4 MSCs (Table 2). Similarly, P40 MSCs exhibited an increase in the expression of the dopaminergic makers DAT and TH, as well as an increase in the expression of MAP2 and Nestin following the DDI (Table 2). Interestingly, P4 and P40 control MSCs also exhibited a slight positive expression of the mature dopaminergic markers TH and DAT, although to a lesser extent than the directly differentiated MSCs (Table 2). 

#### 2.2.2. Immunophenotype following Stage One of the Indirect Dopaminergic Induction

**Figure 6:**
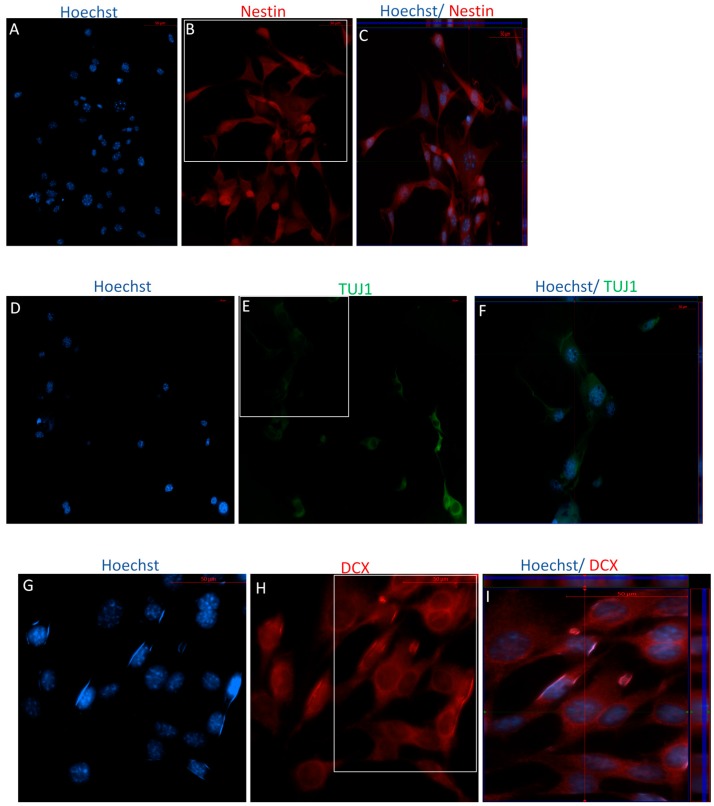
Immunocytochemistry/immunofluorescence (ICC/IF) performed on P4 following Stage One of the IDI. Following exposure to bFGF and EGF, cells revealed positive staining of the immature neuronal markers in P4 cells, including Nestin ((**B**) red), TUJ1 ((**E**) green), and DCX ((**H**) red). Hoechst-labeled P4 NSCs (**A**,**D**,**G**). Orthogonal displays of *z*-stack are represented in merged images (**C**,**F**,**I**).

**Figure 7:**
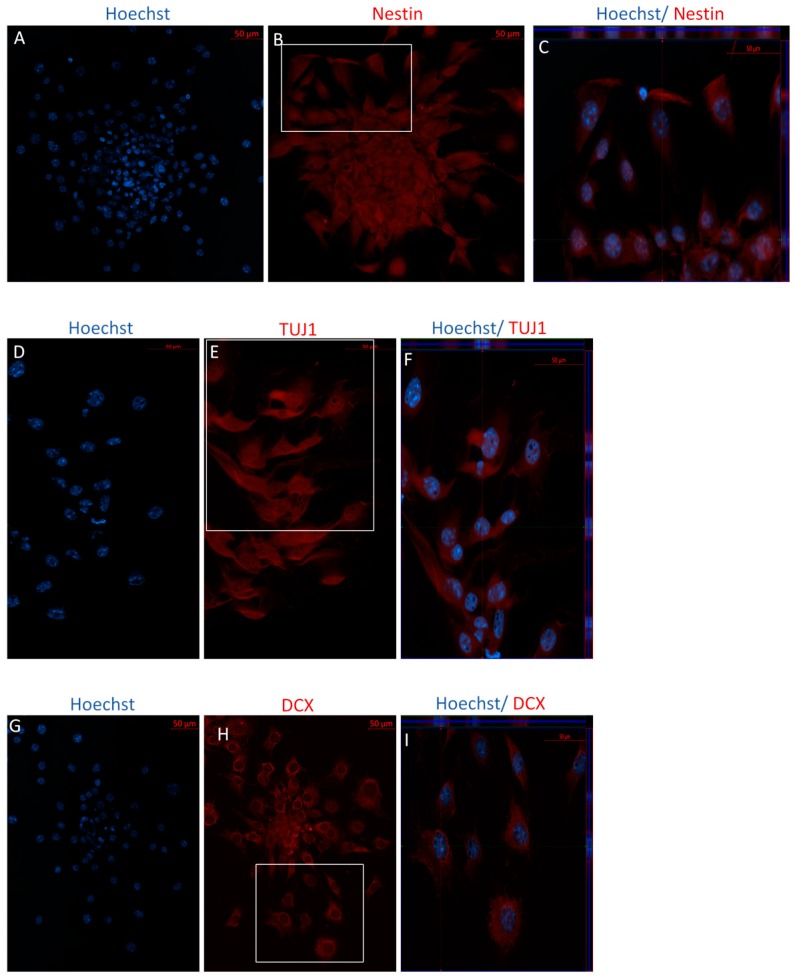
Immunocytochemistry/immunofluorescence (ICC/IF) performed on P40 following Stage One of the IDI. Following exposure to bFGF and EGF, cells revealed positive staining of the immature neuronal markers in P4 cells, including Nestin ((**B**) red), TUJ1 ((**E**) red), and DCX ((**H**) red). Hoechst-labeled P40 NSCs (**A**,**D**,**G**). Orthogonal displays of *z*-stacks are represented in merged images (**C**,**F**,**I**).

Immunocytochemistry analyses following Stage One of the IDI show that both P4 and P40 MSCs expressed the immature neuronal markers doublecortin (DCX), TUJ1, and Nestin, which are indicative of neural stem cells (Figure 6 and Figure 7). Similarly, flow cytometry analyses indicated high levels of expression of these same markers in both early and later passaged MSCs following Stage One of the IDI (Table 3).

#### 2.2.3. Immunophenotype of MSCs following Stage Two of the Indirect Dopaminergic Induction

**Figure 8:**
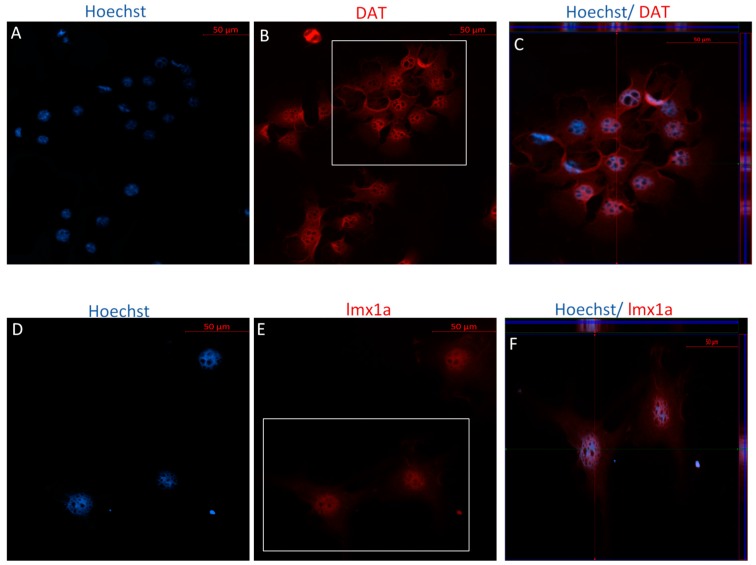
Immunocytochemistry/immunofluorescence (ICC/IF) performed on P4 MSCs following Stage Two of the IDI. Hoechst-labeled P4 MSCs (**A**,**D**) revealed positive staining of the mature dopaminergic marker, DAT ((**B**) red), and the immature dopaminergic marker, LMX1a ((**E**) red), following exposure to SHH, FGF-8, bFGF, and BDNF. There was no bright staining detected for TH for P4 differentiated cells (image not included). Orthogonal displays of *z*-stacks are represented in merged images (**C**,**F**).

**Figure 9:**
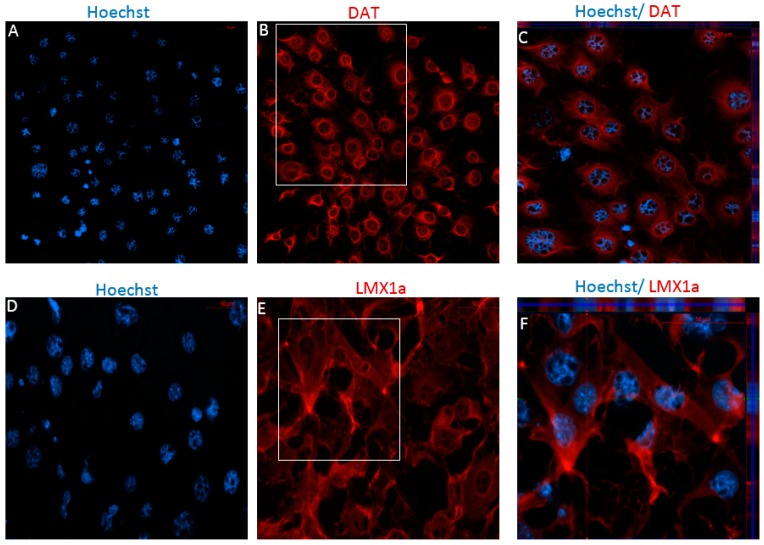
Immunocytochemistry/immunofluorescence (ICC/IF) performed on P40 MSCs following Stage Two of the IDI. Hoechst-labeled P40 MSCs (**A**,**D**) the mature dopaminergic marker, DAT ((**B**) red), and the immature dopaminergic marker, LMX1a ((**E**) red), following exposure to SHH, FGF-8, and BDNF (right). There was no bright staining detected for TH for P40 differentiated cells (image not included). Orthogonal displays of *z*-stacks are represented in merged images (**C**,**F**).

Following Stage Two of the IDI, ICC revealed positive staining for DAT and LMX1a for both P4 and P40 MSCs (Figure 8 and Figure 9). Unlike the MSCs differentiated using the DDI, no bright staining was detected for antibodies against TH for either P4 or P40 MSCs following the IDI (data not shown). Similarly, results from flow cytometry suggest that both early and later passaged MSCs exhibited an increase in the expression of TH following the IDI, relative to control MSCs, although the percent expression was substantially lower than that of P4 and P40 MSCs following the DDI (Table 2). Still, flow cytometry revealed that P4 MSCs exhibited an increase in expression of DAT, Nestin, MAP2, and LMX1a, following the IDI, relative to control MSCs (Table 2). The P40 MSCs also exhibited an increase expression for DAT, Nestin, and LMX1a, relative to control MSCs, following the IDI, but a lower percent expression of MAP2, as compared to P4 MSCs, following the IDI (Table 2).

### 2.3. Upregulation of Dopaminergic-Specific Genes following the Direct and Indirect Dopaminergic Inductions

**Figure 10:**
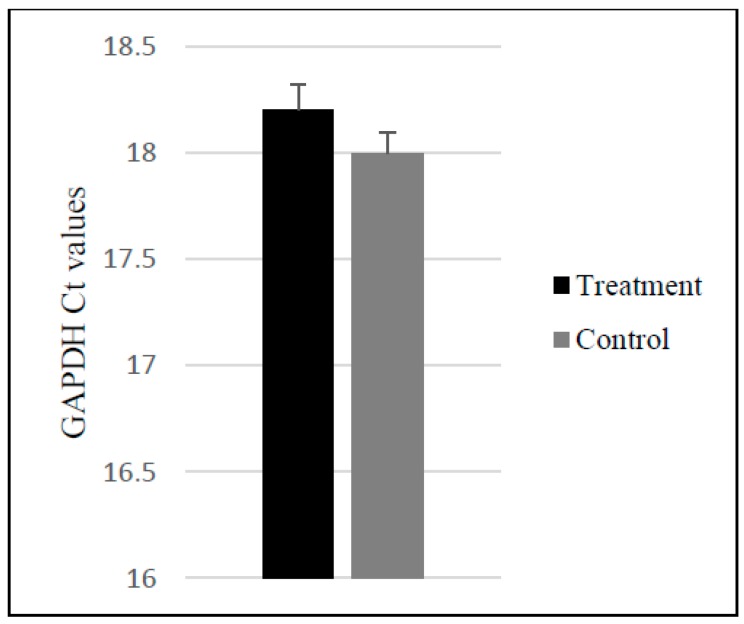
Glyceraldehyde 3-phosphate dehydrogenase (GAPDH) *C*t values were not significantly different between treatment and controls.

**Figure 11:**
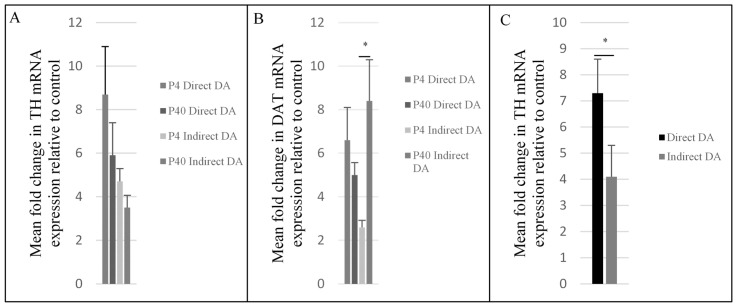
Results from qRT-PCR analyses. (**A**) TH mRNA expression relative to control was not significant. (**B**) DAT mRNA expression relative to control MSCs P40 MSCs exhibited a significant increase in DAT mRNA expression following the IDI as compared to P4 MSCs following the IDI (**C**) MSCs exhibited significantly higher mean fold changes in TH mRNA expression following the DDI as compared to the IDI. Results are presented as mean fold change in mRNA expression ± SEM; * *p* = 0.05.

Quantitative reverse transcriptase-PCR (qRT-PCR) was used to examine the TH and DAT mRNA levels of P4 and P40 MSCs following the DDI and IDI. An independent *t*-test revealed that levels of glyceraldehyde 3-phosphate dehydrogenase (GAPDH) did not significantly differ between control and differentiated MSCs (Figure 10). Following both the DDI and IDI, P4 and P40 MSCs exhibited an upregulation in both TH and DAT relative to controls (Figure 11A,B). Although there were no significant differences in TH mRNA expression, there was a significant difference in DAT mRNA levels between treatment groups *F*(3, 8) = 3.96, *p* = 0.05. Specifically, there was a significant increase in DAT mRNA expression in P40 MSCs following the IDI (M = 8.43 ± 1.9, *p* = 0.04) as compared to P4 MSCs following the IDI (M = 2.63 ± 1.5). Additionally, a 2 × 2 ANOVA demonstrated a significant main effect of induction protocol on TH expression, with P4 and P40 MSCs exhibiting higher mean fold changes of TH mRNA levels following the DDI as compared to P4 and P40 MSCs following the IDI, *F*(1, 8) = 5.2, *p* = 0.05 (Figure 11C). There were no significant main effects of passage number or induction protocol for DAT expression.

### 2.4. Early and Later Passaged MSCs Secrete Dopamine following the Direct and Indirect Dopaminergic Inductions

**Figure 12:**
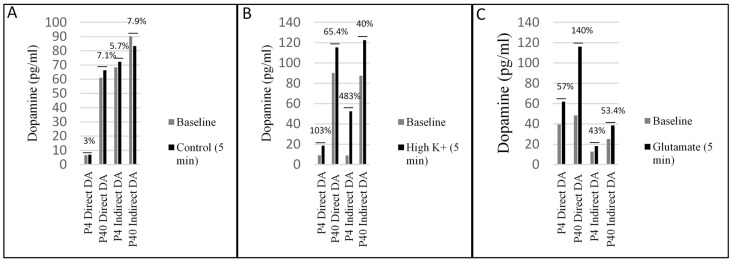
MSCs secrete DA following the DDI and IDI. (**A**) Changes in DA levels (pg/mL) between baseline and control groups. (**B**) Changes in DA levels between baseline and treatment with elevated K^+^. (**C**) Changes in DA levels between baseline and treatment with glutamate. Varying levels of DA were observed across samples at baseline, suggesting that differentiated MSCs spontaneously release DA prior to treatment with K^+^ and glutamate. Because levels of DA were not consistent across all samples at baseline, it is likely that there were different concentrations of DA secreting-MSCs in each of the three wells prior to collection. In order to show effects of K^+^ and glutamate, data is represented as a percent increase from baseline. Results show an increase in expression of dopamine following treatment with elevated K^+^ and glutamate for all treatment groups.

A dopamine ELISA was used to determine if early and later passaged MSCs secrete dopamine following either the DDI or IDI (Figure 12). Samples were collected from three individual wells at baseline from P4 and P40 control MSCs, and P4 and P40 MSCs, following the DDI and IDI. Following baseline collections, MSCs were treated with elevated KCl and glutamate (one well remained untreated to be used as a control) and incubated for 5 min prior to sample collection [57]. Baseline collections from early and later passaged MSCs demonstrated that MSCs spontaneously release dopamine following both the direct and indirect dopaminergic inductions (Figure 12). Levels of dopamine release were increased following treatment with elevated KCl (Figure 12B) and glutamate (Figure 12C), while control levels remained relatively unchanged (Figure 12A). Although dopamine release was observed before stimulation, the increase in dopamine following treatment with KCl and glutamate suggests that these cells release dopamine in a depolarization-dependent manner. Early and later passaged control MSCs did not release dopamine in any detectable levels.

### 2.5. Early and Later Passaged MSCs Exhibit Electrophysiological Activity following the DDI and IDI

**Figure 13:**
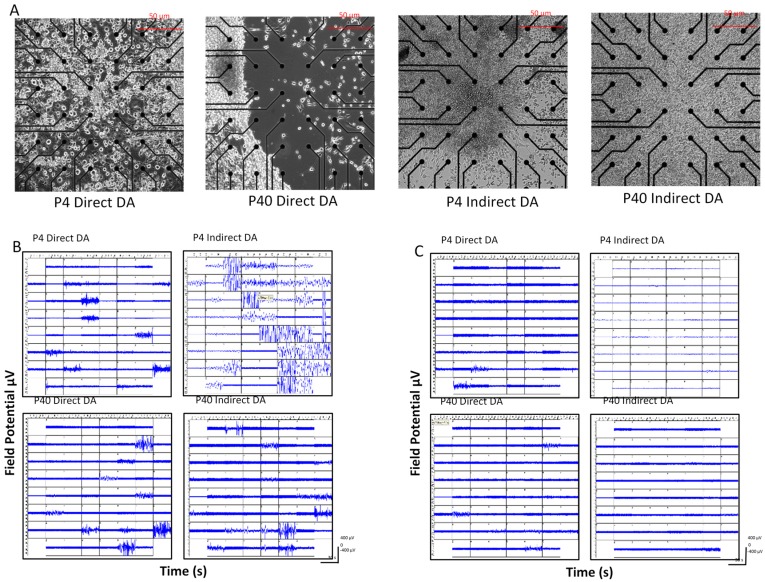
Multi-electrode recordings from early and later passaged MSCs following the DDI and IDI. (**A**) Images of P4 and P40 differentiated MSCs and surrounding electrodes (black dots). (**B**) Recordings of P4 and P40 MSCs following the DDI and IDI. At 2–3 weeks following differentiation, MSCs exhibit spontaneous neuronal activity. (**C**) Neuronal activity is reduced in all groups following addition of TTX to the MEA well at a final concentration of 1 µM. Samples were recorded for 10 min (600 s). TTX was added to culture media at ~300 s.

**Figure 14:**
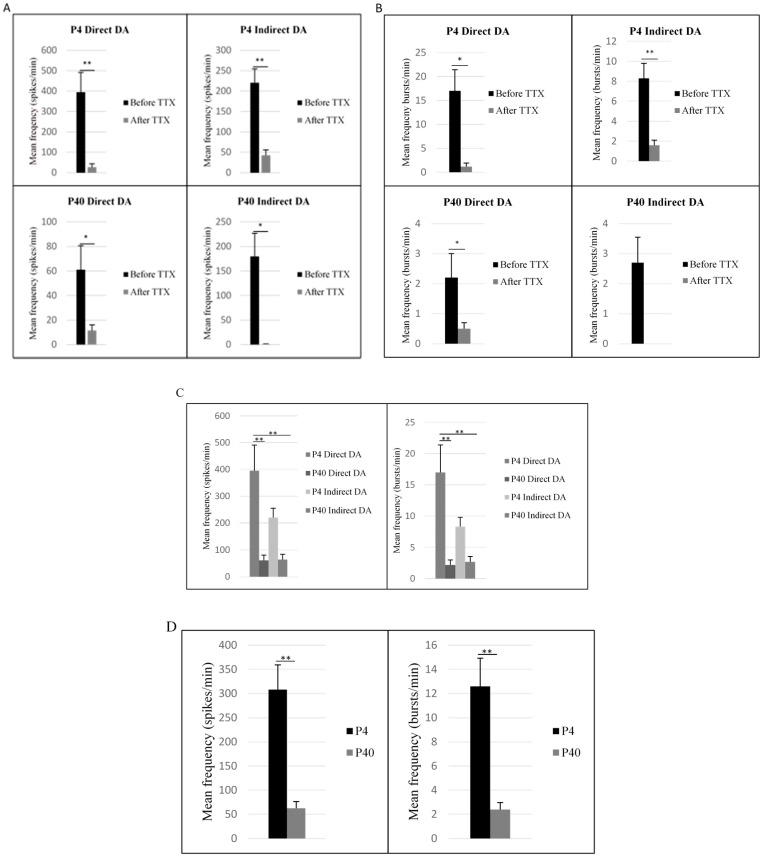
Spiking and bursting activity across all electrodes. (**A**) Spiking frequencies were significantly reduced in all four treatment groups following addition of TTX. (**B**) Bursting frequencies were significantly reduced in P4 MSCs following the direct and indirect inductions, and in P40 MSCs following the DDI. No bursting activity was detected following addition of 1 µM TTX to the MEA well for P40 MSCs following the IDI. (**C**) P4 MSCs exhibited a significant increase in spiking and bursting frequencies following the DDI, as compared to P40 MSCs following both the DDI and IDI. (**D**) P4 MSCs exhibited an increase in spikes and bursts per minute as compared to P40 MSCs following either the DDI or IDI. Results are presented as mean frequencies (spikes or bursts per minute) ± SEM; * *p* < 0.05, ** *p* < 0.001.

Multi-electrode array extracellular recordings were used to measure neuronal activity of early and later passaged MSCs following the DDI and IDI (Figure 13). MEAs were also used to examine the ability of tetrodotoxin (TTX) to silence neuronal activity following differentiation. At 2–3 weeks following direct or indirect differentiation, early and later passaged MSCs exhibited spontaneous neuronal spiking and bursting (Figure 13B). Following the addition of TTX to cell cultures, spiking activity was significantly reduced in all treatment groups (Figure 13C and Figure 14A; P4 DDI; *t*(58) = 3.8, *p* = 0.000321; P40 DDI; *t*(58) = 3.0, *p* = 0.004; P4 IDI; *t*(58) = 6.4, *p* = 2.87 × 10^−8^ P40 IDI; *t*(58) = 3.2, *p* = 0.002). Bursting activity was also significantly reduced following treatment with TTX (Figure 13C and Figure 14B; P4 DDI; *t*(58) = 3.6, *p* = 0.001; P40 DDI; *t*(58) = 2.5, *p* = 0.014; P4 IDI; *t*(58) = 5.8, *p* = 3.15 × 10^−7^). No statistical data was included for the P40 indirect DA group, because bursting activity was completely silenced following addition of TTX (Figure 14B).

Comparisons of neuronal activity before the addition of TTX also demonstrated a significant difference in spiking *F*(2, 232) = 9.0, *p* = 0.000011) and bursting frequencies *F*(2, 232) = 8.4, *p* = 0.000026) between treatment groups (Figure 14C). Specifically, P4 MSCs exhibited an increase in spiking frequency following the DDI (M = 395.5 ± 95.8) as compared to P40 MSCs following the direct (M = 61.1 ± 19.5, *p* = 0.000069) and indirect (M = 63.9 ± 20.1, *p* = 0.000081) inductions. Similarly, P4 MSCs exhibited an increase in bursting frequency following the DDI (M = 17 ± 4.4) as compared to P40 MSCs following the DDI (M = 2.7 ± 0.85, *p* = 0.000092) and IDI (M = 2.2 ± .8, *p* = 0.000187). There were no significant differences in spiking or bursting frequencies between P4 MSCs following either the DDI or IDI. Additionally, a two-way ANOVA demonstrated a main effect of passage number on both spiking and bursting activity. P4 MSCs exhibited significantly higher spiking *F*(1, 232) = 21.6, *p* = 0.000006 and bursting frequencies *F*(1, 232) = 18.5, *p* = 0.000025 as compared to P40 MSCs following either the DDI or IDI (Figure 14D). There were no significant main effects of induction protocol for spiking or bursting activity.

## 3. Discussion

In the current study, we investigated the influence of different induction methods (DDI vs. IDI), as well as serial passaging of MSCs (early vs. later passaged MSCs), in order to identify an optimal method for the generation of dopaminergic neurons from MSCs to be used in PD research. Here, we have shown that early passaged bone marrow-derived MSCs are most efficient at producing dopaminergic-like cells following a DDI.

### 3.1. Morphological Changes and Expression of Neuronal and Dopaminergic Markers

Following characterization, MSCs exhibited a change in morphology toward a neuronal phenotype, along with changes in the expression of neuronal and dopaminergic markers in both early and later passaged MSCs using either the DDI or IDI. In the past, studies have utilized chemical induction protocols for the generation of neuronal-like cells from MSCs and have described similar changes in cellular morphology [72,73,74,75]. Still, it has been suggested that these alterations are due to cellular toxicity and cell shrinkage in response to environmental stress, as opposed to true neurite outgrowth [64]. Specifically, exposure to chemicals, such as dimethyl sulfoxide/butylated hydroxyanisole (DMSO/BHA) and isobutylmethylxanthine/dibutyryl cyclic AMP (IBMX/dcAMP), have been said to lead to the death and rapid disruption of the actin cytoskeleton and retraction of selected areas of the cell perimeter and cytoplasm [52,64,76,77]. Additionally, Tao and colleagues [78] demonstrated that IBMX and DMSO were unable to induce sustained neuronal differentiation of MSCs as compared to MSCs exposed to cytokines, such as bFGF, EGF, and platelet-derived growth factor (PDGF), which induced neuronal morphology in MSCs and the expression of neuronal markers for up to three months. Similarly, Khoo and colleagues [64] explained that the observed changes in the morphology of MSCs following a cytokine-induced induction are the result of regulated steps that involve true outgrowth and motility of cellular extensions, and not of cellular toxicity resulting from chemical-induction. The present study utilized growth factors and morphogens, including: (1) SHH and FGF-8, which interact during development to create induction sites for dopaminergic neurons [57,78,79,80,81,82]; (2) bFGF, which is involved in the development and maintenance of the nervous system, as well as the enhancement and survival of dopaminergic neurons [83,84,85,86,87]; and (3) BDNF, which plays a critical role in promoting the survival and differentiation of midbrain dopaminergic neurons [88,89,90,91,92]. Therefore, it is highly likely that the observed morphological changes, and increased expression of neuronal and dopaminergic markers following treatment with SHH, FGF-8, and BDNF, represent true cellular differentiation toward a neuronal phenotype.

### 3.2. Effects of Passage Number on Dopaminergic Differentiation

This study investigated the influence of serial passaging on the ability of MSCs to differentiate into dopaminergic neurons. Previously, researchers have demonstrated that early passaged MSCs exhibit myogenic and neurogenic markers, prior to differentiation, potentially explaining why MSCs have the capacity to differentiate into multiple cell lineages [50,75,93,94,95,96]. However, this does not indicate that increased passaging will compromise the ability of undifferentiated MSCs to ameliorate deficits in animal models of degeneration. In fact, Rossignol and colleagues [97] demonstrated that later passaged undifferentiated mouse BM-MSCs were better able to delay cognitive and motoric deficits following transplantation into the R6/2 mouse model of Huntington’s disease (HD) as compared to early passaged MSCs. Therefore, increased passaging may influence the ability of MSCs to differentiate into cells of a neuroectodermal lineage, but their ability to provide trophic support is likely preserved at higher passages.

Based on findings of passaging and subsequent differentiation potential, we hypothesized that early passaged MSCs would express neuronal markers prior to differentiation, and there would be an increase in these markers following neural induction. We also anticipated that this expression would gradually decline at later passages, resulting in a lower plasticity for neural induction. Our results show that: (1) P4 and P40 MSCs exhibited positive expression of neuronal markers prior to induction (Table 1 and Table 2); (2) P4 and P40 MSCs expressed a high percentage of immature neuronal markers (i.e., Nestin, TUJ1, DCX) following Stage One of the IDI (Figure 6 and Figure 7), as well as an increase in the expression of neuronal and dopaminergic markers following both dopaminergic inductions (Table 2; Figure 4, Figure 5, Figure 8 and Figure 9); (3) P4 and P40 differentiated MSCs exhibited an increase in DAT and TH mRNA levels as compared to control MSCs (Figure 11A,B); and (4) P4 and P40 MSCs secreted dopamine in a depolarization-dependent manner following the DDI and IDI (Figure 12). Similarly, studies have shown that later-passaged MSCs cultures were still able to undergo morphological and gene expression changes toward neuronal-like cells following exposure to cytokines [64].

Although increased passaging may not compromise the expression of neuronal and dopaminergic markers, or the ability of differentiated MSCs to secrete dopamine, it appears that passage number does influence electrophysiological properties of differentiated MSCs. Specifically, it was observed that P4 MSCs exhibited a significant increase in spiking and bursting frequencies as compared to P40 MSCs following the DDI and IDI (Figure 14D). In a study conducted by Cepeda and colleagues [98], researchers compared the electrophysiological characteristics of young and aged neostriatal neurons using in vitro slice preparations. From their results, it was determined that major electrophysiological characteristics of neostriatal neurons are altered during aging. Specifically, researchers discovered that changes in electrophysiological properties of aged neostriatal neurons resulted in decreases in cellular excitability [98]. Additionally, it has been reported that even limited passaging (6–12 passages) of neural precursor cells alters neuronal network functionality, leading to decreases in the percentage of active neurons, bursting frequency, and synchronicity [99]. Therefore, previous reports of rapid aging and accelerated senescence following sustained passaging may explain why P40 MSCs exhibit a decrease in excitability as compared to P4 MSCs. Overall, these results indicate that early passaged MSCs more efficiently differentiate into neuronal-like cells as compared to later passaged MSCs. Moreover, in situations that necessitate in vitro expansion (i.e., clinical applications), the use of higher passaged MSCs should be met with caution.

### 3.3. Effects of Induction Protocol on Dopaminergic Differentiation

Previous studies have reported a 46.7–67% efficiency of dopaminergic induction of BM-MSCs following direct exposure to SHH and FGF-8 [57,61]. Additionally, Trzaska and colleagues [100] demonstrated that induced MSCs released DA upon depolarization and express characteristics of functional neurons as evidenced through electrophysiology. In comparison, studies utilizing an indirect approach report that only 12.7–35% of induced cells express TH following differentiation [54,55]. Additionally, these studies do not discuss if MSCs secrete dopamine or express electrophysiological properties following differentiation. Alternatively, Dezawa and colleagues [48] reported a 41% efficiency of TH-expressing cells following an indirect induction. MSCs were shown to secrete dopamine in a depolarization-dependent manner following treatment with glial cell-line derived neurotrophic factor (GDNF) and exhibited action potentials following exposure to BDNF [48]. However, that study did not utilize SHH and FGF-8, which are required for dopaminergic neuron induction during development. Instead, MSCs were transfected with Notch intracellular domain (NCID) to induce neuronal differentiation of MSCs [48,57]. Due to these discrepancies, we were interested in comparing different cytokine-based induction methods to identify the most effective process for obtaining dopaminergic neurons from MSCs.

In the present study, MSCs were cultured for 12 days in dopaminergic induction media, with half media changes every 3 days. Trzaska and colleagues [100] state that changes in media lead to the removal of endogenous factors that are important for dopaminergic maturation. Here, we found that half media changes were necessary to keep the cells healthy and replenish nutrients while not completely removing endogenous factors by performing full media changes. From our results, we observed that both DDI and IDI protocols led to the generation of cells that positively express both neuronal and dopaminergic markers, as evidenced through ICC and flow cytometry. Results from qRT-PCR and flow cytometry also indicated that both directly and indirectly differentiated MSCs exhibited an increase in the expression of TH and DAT mRNA as compared to undifferentiated MSCs. Still, results from flow cytometry suggested a substantially lower percent expression of TH in MSCs following the IDI as compared MSCs following the DDI (Table 2). This decreased TH expression was further confirmed by the lack of bright staining of TH in indirectly differentiated MSCs using ICC (Figure 8 and Figure 9). Additionally, qRT-PCR analyses revealed that MSCs differentiated using the DDI exhibited a significantly higher mean fold change in TH mRNA levels as compared to MSCs differentiated using the IDI. Because positive expression of TH (i.e., the rate-limiting enzyme in dopaminergic production) is considered the gold standard for the identification of dopaminergic neurons, it would appear that the DDI is a more efficient method for generating DA-neurons from MSCs, as compared to the IDI. Nevertheless, the lower TH levels observed following the IDI did not affect the ability of these cells to secrete dopamine or elicit electrophysiological responses.

Still, these results suggest that it may be unnecessary to first direct MSCs toward a neural stem cell-like lineage prior to terminal dopaminergic differentiation. Although exposure to bFGF and EGF resulted in the high positive expression of the immature neuronal markers TUJ1, Nestin, and DCX, in P4 and P40 MSCs (Table 3; Figure 6 and Figure 7), undifferentiated MSCs already exhibited a relatively high percent expression of TUJ1, and Nestin (Table 1), prior to induction. Thus, it appears that the examined increase in immature neuronal markers between undifferentiated and neurally induced MSCs has little influence on the ability of MSCs to differentiate into dopaminergic neurons. However, the higher expression of immature neuronal markers following Stage One may explain the lower expression of TH following Stage Two of the IDI. In other words, the higher percentage of immature neuronal markers expressed in MSCs following Stage One of the IDI, as compared to undifferentiated MSCs, may be responsible for keeping the cells in a more immature-like state, resulting in lower expression of TH following terminal dopaminergic differentiation.

Importantly, MSCs also continued to exhibit relatively high levels of the immature neuronal markers Nestin (Table 2) and TUJ1, the immature dopaminergic marker LMX1a (Table 2; Figure 4, Figure 5, Figure 8 and Figure 9), and positive DAT and TH expression, following both the DDI and IDI (Table 2; Figure 4, Figure 5, Figure 8 and Figure 9). The concomitant expression of immature neuronal and dopaminergic markers with TH and DAT and spontaneous neuronal activity suggests that these cells are between the immature and mature dopaminergic states. 

Previous studies have shown that various differentiation stages of dopaminergic progenitors and neurons survive and restore motor deficits following transplantation [101,102,103]. Qiu and colleagues [103] conducted a study to determine the optimal stage of differentiation for transplantation by comparing engraftment efficacy, survival, differentiation, and maturation of dopaminergic progenitors, immature dopaminergic neurons, and mature dopaminergic neurons. Their results indicate that: (1) all stages of dopaminergic cells are able to survive following transplantation; (2) dopaminergic progenitors are less effective at restoring behavioral deficits due to their lack of maturation following transplantation; and (3) immature dopaminergic neurons produce the highest percentage of NeuN and TH positive cells, in vivo. Many researchers have concluded that both mature and immature dopaminergic neurons efficiently survive and differentiate in vivo [103]. Therefore, differentiated MSCs from this study appear to be ideal candidates for transplantation due to the positive expression of immature and mature neuronal and dopaminergic markers following induction.

Overall, both the DDI and IDI effectively produce dopaminergic-like cells that are suitable for transplantation. The positive expression of immature-neuronal markers in undifferentiated MSCs may be responsible for the ability of these cells to differentiate into cells of an ectodermal lineage and negates the need for neuronal differentiation prior to dopaminergic induction. Therefore, the DDI appears to be more efficient for dopaminergic differentiation of MSCs as compared to the IDI.

### 3.4. Co-Expression of TH and DAT

As mentioned above, positive expression of TH is a major determining factor for the presence of dopaminergic neurons, because it is the enzyme that converts l-tyrosine to l-3,2-dihydroxyphenylalanine (l-DOPA) [104,105]. In addition to dopamine, TH is also a marker of all catecholamine (CA)-containing neurons, including epinephrine, and norepinephrine, and is therefore expressed in adrenergic and noradrenergic neurons, as well as in dopaminergic neurons [106,107,108,109]. Because of this common expression of TH, it is necessary to specifically distinguish dopaminergic neurons from other CA neurons using an additional marker.

In the present study we examined the expression of the dopamine transporter DAT following our differentiation protocols, in addition to characterizing the expression of TH. In vertebrates, DAT is exclusively expressed in dopaminergic neurons. The distribution of DAT mRNA-containing cells has been identified to be more restricted to the substantia nigra pars compacta and the ventral tegmental area (VTA), while TH mRNA-containing cells have been detected in the hypothalamus, midbrain, and pons, with the highest levels being detected in the substantia nigra and VTA [104,106,107,110,111,112,113,114,115,116,117]. Because our findings indicate positive expression of both TH and DAT following the dopaminergic differentiation protocols, we are confident that the resulting cell types are in fact dopaminergic.

Nevertheless, these cells should be further characterized to differentiate between A9 dopaminergic neurons of the substantia nigra and A10 dopaminergic neurons of the VTA [118,119,120]. A9 neurons are specifically degenerated in patients with Parkinson’s disease and have been shown to be a critical component of intrastriatal grafts for recovery in motor performance in rodent models of PD. Differentiated cells can be characterized for the expression of G-protein-regulated inward-rectifier potassium channel 2 (GIRK2), a protein that specifically identifies the A9-subtype of dopaminergic neurons, and calbindin proteins, which are broadly used to identify A10 populations [11,119,121]. Future transplantation studies should characterize cells for the expression of GIRK2 and calbindin to ensure successful reintegration into the host striatum.

## 4. Materials and Methods

### 4.1. Isolation and Expansion of MSCs

Two adult male Sprague Dawley rats (350–400 g) were euthanized via inhalation of 80% carbon dioxide and 20% oxygen following procedures of an animal use protocol approved by the Institutional Animal Care and Use Committee (IACUC; approval # 14-30, approved on 26 November 2014) at Central Michigan University (CMU). The bone marrow was extracted from the tibia and femur by drilling the bone with an 18-gauge needle and removing the bone marrow with a syringe. Following extraction, the bone marrow was suspended in MSC medium, consisting of Minimum Essential Medium Alpha Modification (α-MEM: Life Technologies, Carlsbad, CA, USA), 10% fetal bovine serum (FBS; Life Technologies), 10% horse serum (HS; Life Technologies), and 1% penicillin-streptomycin (Sigma, St. Louis, MO, USA), and centrifuged. The number of cells was determined using a hemocytometer, and plated in a 25-cm^2^ tissue culture plate (TPP MidSci, St. Louis, MO, USA). The flask was incubated at 37 °C, 5% CO_2_ until the cells grew to ~80% confluency and were then passaged. To complete the passaging process, cells were detached from the flask using 0.25% Trypsin-EDTA (Life Technologies) and re-plated at a density of 2 × 10^5^ cells/25-cm^2^ flask. This procedure was repeated an additional 3 times to achieve a population of early passage (P4) MSCs and an additional 39 times for later passage (P40) MSCs.

### 4.2. Direct Dopaminergic Induction (DDI)

MSCs underwent the DDI using a modified version of the protocol described by Trzaska and colleagues [57]. Briefly, MSC media was removed from P4 and P40 MSCs and replaced with dopaminergic induction media, containing Neubrobasal™ Media (Life technologies) 2% GIBCO^®^ B-27^®^ Supplement (Life technologies), 250 ng/mL sonic hedgehog (SHH; R&D Systems, Minneapolis, MN, USA), 100 ng/mL fibroblast-growth factor-8 (FGF8; R&D Systems), and 25 ng/mL basic fibroblast growth factor (bFGF; Life technologies). Half of the media was changed every 3 days, and cells were incubated for a total of 9 days. After 9 days, 50 ng/mL BDNF (Peprotech, Rocky Hill, NJ, USA) were added to the culture dishes, and the cells were incubated for an additional 3 days before characterization. Undifferentiated MSCs were used as control cells for both the DDI and IDI protocols. These cells remained in culture for the duration of the dopaminergic inductions but did not receive any growth factors or morphogens.

### 4.3. Indirect Dopaminergic Induction (IDI)

#### 4.3.1. Stage One

P4 and P40 MSCs that underwent the IDI were first directed toward a neuronal lineage prior to dopaminergic induction. Briefly, when the cells reached passages 4 or 40, they were re-plated in 3 × 25-cm^2^ flasks at a density of 5 × 10^5^ and treated with neural induction media that consisted of Dulbecco’s Modified Eagle Medium: Nutrient Mixture F-12. (DMEM/F12; Life Technologies), 2% GIBCO^®^ B-27^®^ Supplement (Life Technologies), 20 ng/mL bFGF (Life Technologies), and 20 ng/mL EGF (Life Technologies). At 48–72 h following initial treatment with neural induction media, clusters of cells (i.e., neurospheres) were observed suspended in the media. One week after the initial plating, neurospheres were subcultured using Accutase (Sigma) to promote the dissociation of the spheres into a single cell suspension. Cells were centrifuged at 200 g for 5 min, re-suspended in fresh neural induction media, and re-plated into the original 25-cm^2^ flasks. Neurospheres were expanded for an additional 2 weeks (1 passage/week) to ensure a sufficient number of cells for all future experiments. At the end of Stage One, approximately 5 × 10^6^ neurospheres were collected for characterization using ICC and flow cytometry analyses. The remaining cells underwent Stage Two of the IDI.

#### 4.3.2. Stage Two

Following Stage One of the IDI, neurospheres were plated on poly-l-ornithine-coated flasks and allowed to adhere overnight. The next day, the neural induction media was removed and cells were treated with the dopaminergic induction media. As previously described, half of the media was removed and replenished with growth factors every 3 days for a total of 9 days. After 9 days in culture, 50 ng/mL BDNF was added to the culture dishes, and cells were incubated for an additional 3 days before characterization.

### 4.4. Immunocytochemistry/Immunofluorescence

P4 and P40: control MSCs, neurospheres, and differentiated MSCs were fixed with 4% paraformaldehyde (PFA) and blocked with a solution of phosphate buffered saline (PBS), 0.1% Triton™ X-100 (Sigma), and 10% normal goat serum (NGS; Sigma). All samples were stained with primary antibodies diluted in PBS, 0.1% Triton X-100. Antibodies and dilutions used for labelling neurospheres from Stage One of the IDI were as follows: rabbit anti-DCX polyclonal IgG, 1:500; mouse anti-TUJ1 monoclonal IgG, 1:500; and rabbit anti-Nestin polyclonal IgG, 1:250 (Abcam, Cambridge, MA, USA). Following the DDI and IDI, samples were stained to detect the following markers: rabbit anti-TH polyclonal IgG, 1:1000; rabbit anti-DAT polyclonal, 1:200; and rabbit anti-LMX1a, 1:200 (Abcam). The samples with primary antibodies were stored at 4 °C overnight. The following day, all samples were rinsed twice with PBS, 0.1% Triton X-100, stained with the secondary antibody targeting the species that the primary was generated in, either Alexa Fluor^®^ 488 goat anti-mouse IgG or Alexa Fluor^®^ 594 goat anti-rabbit (Life Technologies) at a 1:300 dilution, and incubated at room temperature for 1 h. The samples were then rinsed twice with PBS, counterstained with Hoechst (Life Technologies) in PBS with 0.1% Triton X-100 (at a 1:1000 dilution), and rinsed three times in PBS. All samples were mounted with Fluoromount™ aqueous mounting media (Sigma) and stored in 4 °C until they were analyzed using a Zeiss Axio fluorescent microscope. Four sections were captured from all images at 2 μm intervals to generate z-stacks. Orthogonal displays of z-stacks are represented in merged images in Figure 4, Figure 5, Figure 6, Figure 7, Figure 8 and Figure 9.

### 4.5. Flow Cytometry

#### 4.5.1. Extracellular Staining

P4 and P40 undifferentiated MSCs were counted and plated in a 96 U-bottom well plate (MidSci) at a density of 1.5 × 10^5^–2.0 × 10^5^ per well. Samples were washed twice with 100 μL in PBS containing 1% bovine serum albumin (BSA; Sigma) and 0.1% sodium azide (Sigma), and centrifuged at 2000 rpm for 1 min. Undifferentiated MSCs were stained with primary antibodies at the following concentrations: rabbit anti-CD90 monoclonal IgG, 1:50; mouse anti-CD105 monoclonal IgG, 1:500; rat anti-CD34 mouse monoclonal IgG; 1:200; and anti-MHC class II monoclonal IgG, 1:500 (Abcam). Cells were incubated on ice for 45 min and centrifuged at 2000 rpm for 1 min. Following incubation, cells were washed one time in PBS/1% BSA/0.1% sodium azide and then re-suspended in secondary antibody (Alexa Fluor^®^ 488 goat anti-rabbit IgG, Alexa Fluor^®^ 488 goat anti-mouse IgG, or Alexa Fluor^®^ 488 goat anti-rat IgG; Life Technologies) at a 1:300 dilution. Samples were incubated on ice for 30 min in the dark. Following secondary staining, samples were washed an additional three times and fixed with 4% paraformaldehyde for 10 min on ice. The cells were re-suspended in PBS/1% BSA/0.1% sodium azide and stored at 4 °C until flow cytometry analysis.

#### 4.5.2. Intracellular Staining

For the intracellular staining of samples, cells were permeabilized with 0.1% Saponin (Sigma) for 10 min on ice prior to antibody staining. Following Stage One of the IDI, P4 and P40 neurospheres were dissociated with Accutase (Sigma), plated in a 96 U-bottom well plate at a density of 1.5 × 10^5^–2.0 × 10^5^ per well, and stained with the following primary antibodies: rabbit anti-DCX polyclonal IgG, 1:500; mouse anti-TUJ1 monoclonal IgG, 1:50; and rabbit anti-Nestin polyclonal IgG, 1:250 (Abcam). Following the DDI and IDI, differentiated and control cells were treated with Accutase (Sigma) to promote detachment from the flask surface and plated in a 96 U-bottom well plate at a density of 1.5 × 10^5^–2.0 × 10^5^ per well. Cells from both inductions and control MSCs were stained with the following primary antibodies: rabbit anti-TH polyclonal IgG, 1:500; rabbit anti-DAT polyclonal, 1:200; rabbit anti-MAP2, 1:500, rabbit anti-LMX1a polyclonal IgG, 1:200, and rabbit anti-Nestin polyclonal IgG, 1:250. All samples were incubated on ice for 45 min, rinsed with PBS/1% BSA/0.1% sodium azide, and resuspended in secondary antibody (Alexa Fluor^®^ 488 goat anti-rabbit IgG, Alexa Fluor^®^ 488 goat anti-mouse IgG, or Alexa Fluor^®^ 488 goat anti-rat IgG; Life technologies) at a 1:300 dilution, and incubated on ice, in the dark, for 30 min. Following secondary staining, samples were washed an additional three times and fixed with 4% paraformaldehyde for 10 min on ice. The cells were re-suspended in PBS/1% BSA/0.1% sodium azide and stored at 4 °C until flow cytometry analysis.

#### 4.5.3. Flow Cytometry Analysis

Prepared samples were placed on ice and analyzed using the BD FACSAria flow cytometer (BD Bioscience, San Jose, CA, USA) and BD FACSdiva software. Prior to the flow cytometry analyses, the samples were re-suspended in PBS, transferred to plastic falcon tubes, and vortexed for 10 s. Unstained samples were loaded first, followed by the negative control samples from each experimental group to set the scatter gate plot and fluorescein isothiocyanate (FITC) threshold. The scatter gate plot was established using forward scatter (FCS) and side scatter (SSC) emissions, and set to exclude any debris. The FITC threshold was set to include 1% of the cells from the gated scatter plot. Ten thousand cells were analyzed from each individual sample, and the BD FACSdiva software reported the percentage of gated cells that cross the FITC threshold.

### 4.6. Reverse Transcriptase-Polymerase Chain Reaction

Following the dopaminergic induction protocols, media from the DDI, the IDI, and control MSCs (P4 and P40 MSCs were analyzed for all groups) were removed and cells were washed two times with PBS. Cells were incubated with 2 mL TRIzol^®^ reagent (Life Technologies) per flask, for five minutes, to completely lyse the cells. Lysates were stored at −80 °C until RNA isolation was performed. RNA was isolated from the lysate following the manufacturer’s protocol (Life Technologies). Briefly, samples were thawed and incubated at room temperature for five minutes. Cells were homogenized by adding 0.2 mL of chloroform to each 1 mL of TRIzol^®^ reagent, and the samples were mixed for 15 s and incubated for 2–3 min at room temperature. Samples were vortexed, and the aqueous phase of the samples were removed and placed into new tubes, following centrifugation. Samples were incubated in 100% isopropanol (Sigma) for 10 min, followed by centrifugation for an additional 10 min. The supernatant was removed from the tube, and the remaining RNA pellet was washed with 75% ethanol (Sigma), vortexed for 10 s, and centrifuged. The ethanol wash was discarded, and the RNA pellet was allowed to air dry for 20–30 min before resuspension in RNase-free water (Qiagen, Valencia, CA, USA).

Following RNA isolation, the iScript Advanced cDNA Synthesis Kit for RT-qPCR (Biorad, Hercules, CA, USA) was used to transcribe complimentary DNA (cDNA) from the RNA. The cDNA was utilized in a series of RT-PCR reactions to quantify mRNA expression levels of certain genes, specifically, TH (forward primer: 5′-ACTGTGGAATTCGGGCTATG-3′; reverse primer: 5′-TGCTGTGTCTGGGTCAAAG-3′) and DAT (forward primer: 5′-CCTCCATTAACTCCCTGACAAG-3′; reverse primer: 5′-CATTGTGCTTCTGTGCCATG-3′). The qRT-PCR reactions were performed in triplicates and repeated two times. Each reaction consisted of 1 µL diluted cDNA, 0.5 µL of the 20 µM reverse and forward primers, and 10 µL iQ™ SYBR^®^ Green Supermix (Biorad). Reactions were loaded into 92-well RT-PCR plates (MidSci) and centrifuged at 4000 RPM for 5 min at 4 °C to uniformly distribute each reaction within all of the wells. For RT-PCR reactions, the samples were placed in a thermal cycler and incubated at 95 °C for ten minutes to activate the polymerase enzyme. Next, they underwent 40 cycles of a denaturing stage (95 °C for 15 s) and an annealing stage (60 °C for 1 min), during which time the camera was set to record.

### 4.7. Enzyme-Linked Immunosorbent Assay

Enzyme-linked immunosorbent assays were used to measure dopamine release from differentiated MSCs. Undifferentiated P4 and P40 MSCs and neurospheres were plated on poly-l-ornithine coated coverslips in 6-well plates (3 wells/group) prior to differentiation. P4 and P40 control MSCs were also quantitated for dopamine release. Following the DDI and IDI, dopaminergic induction media was removed, and cells were replenished with Hanks Balanced Salt Solution (HBSS) and incubated in a humidified chamber at 37 °C for 30 min prior to sample collection. Samples were collected from three separate wells; one well remained untreated as a control (well 1), the second well was treated with elevated KCl (K^+^) (well 2), and the third well was treated with glutamate (well 3). Baseline collections (two 120 µL samples from each well) were taken prior to treatment with elevated KCl (56 mM) and glutamate (100 µM). Cells were then incubated for 5 min at 37 °C, at which time additional samples (two 120 µL samples from each well) were collected. Samples were stored at −80 °C until dopamine was measured with a Dopamine Research ELISA (Labor Diagnostika Nord, Nordhorn, Germany). According to the manufacturer, the analytical sensitivity of the DA ELISA kit is 3.3 pg/mL. The ELISA was performed according to the manufacturer’s instructions and all samples were run in duplicates. Dopamine concentrations were determined using a spectrophotometer with the absorbance set to 450 nm.

### 4.8. Multi-Electrode Array

Electrophysiological activity was measured using multi-electrode arrays (MEAs). Standard 1-well MEAs (60 electrodes, 59 recording electrodes, and one internal ground electrode in an 8 × 8 grid arrangement) were used for all experiments. Prior to plating, MEAs were autoclaved and treated with sterile FBS for 30 min at room temperature. Following hydrophilic treatment, a drop of 0.1% polyethyleneimine (PEI) was added directly to the electrode area and incubated for 30 min at 37 °C, rinsed 4× with H_2_O, and allowed to dry. A final coating of 50 µg/mL laminin, diluted in Neurobasal Medium, was applied to the electrode surface for 30 min at 37 °C. Undifferentiated P4 and P40 MSCs were plated in two MEAs at a density of 5 × 10^4^ cells/10 µL MSC media onto the laminin coated surface. Similarly, P4 and P40 neurospheres from Stage One of the IDI were plated onto two MEAs at a density of 15–20 neurospheres/10 µL neural induction media. Cells were allowed to adhere for 30–45 min before 500 µL–1 mL media was added to each well. The following day, media was removed and replaced with dopaminergic induction media. As previously described, cells were incubated for 9 days before BDNF was added to the media. Cells were cultured for an additional 5–12 days for a total of 2–3 weeks, with half of the media being removed and replaced every 3 days. Extended differentiation was necessary because cultures on MEAs take 2–3 weeks to reach a steady state of activity [122]. Following differentiation, MEAs were transferred to the recording preamplifier. TTX at a final concentration of 1 µM was added to culture media during recording. All recordings were done using an MEA2100 Lite head stage and amplifier with a sample rate of 10,000 Hz. Neuronal activity was analyzed using MC-Rack software (MultiChannel Systems) and NeuroExplorer (Version 5, Madison, AL, USA). Spikes were counted when the extracellular recorded signal exceeded 9 standard deviations of the baseline noise.

### 4.9. Statistical Analysis

Relative mRNA expression was analyzed using the comparative threshold cycle (*C*_t_) method or the 2^−ΔΔ*C*t^ method, which presents the fold change in relative gene expression between a control and treatment sample. First, mRNA from each sample (i.e., P4 and P40 control MSCs, P4 and P40 DDI, and P4 and P40 IDI) was normalized to a house keeping gene, glyceraldehyde 3-phosphate dehydrogenase (GAPDH), one of the most commonly used reference genes in RT-PCR [123], to obtain Δ*C*_t_ (Δ*C*_t_ = (*C*_t_ gene of interest − *C*_t_ internal control)). ΔΔ*C*_t_ was obtained by subtracting the Δ*C*_t_ of treated samples− Δ*C*_t_ of control samples (ΔΔ*C*_t_ = [(*C*_t_ gene of interest (treatment) − *C*_t_ internal control (treatment)) – (*C*_t_ gene of interest (control) − *C*_t_ internal control (control))]. Finally, the fold change due to treatment was determined by raising 2 to the negative ΔΔ*C*_t_ (2^−ΔΔ*C*t^) [124].

One-way ANOVAs were used to compare mean fold changes in TH and DAT mRNA expression between treatment groups. Additionally, a two-way ANOVA was used to compare levels of mRNA expression of each gene of interest between early and later passaged MSCs following the DDI and IDI. MEA data is presented as mean frequencies of spikes and bursts per minute. Paired sample *t*-tests were used to compare changes in spiking and bursting frequencies before and after TTX. One-way ANOVAs were used to compare spiking and bursting frequencies between treatment groups. A two-way ANOVA was used to compare spiking and bursting frequencies between early and later passaged MSCs following the DDI and IDI. Data are presented as mean ± SEM. All statistical tests were performed in SPSS (IBM). In all statistical analyses, *p* ≤ 0.05 was considered significant. Pairwise comparisons were conducted using Tukey HSD post-hoc tests when applicable.

## 5. Conclusions

Mesenchymal stem cells have the potential to differentiate into dopaminergic-like cells following either a DDI or IDI, in vitro. However, our findings suggest that early passaged MSCs that have undergone the DDI are more efficient at generating neuronal, and more specifically, dopaminergic-like cells as compared to later passaged MSCs or MSCs that have undergone the IDI. The expression of neuronal and dopaminergic markers, as well as the ability of these cells to secrete dopamine and elicit spontaneous neuronal activity, make them a promising cell source for transplantation studies. Future studies will identify if these cells are able to survive and reintegrate into the host striatum and ameliorate the motoric deficits associated with PD.

## Figures and Tables

**Table 1:** Flow cytometry analysis of bone marrow derived mesenchymal stem cells (MSCs).

Marker	P4 MSCs	P40 MSCs
CD90	965	36.2%
CD105	1.5%	8.5%
MHC II	<0.1%	<0.1%
CD34	<0.1%	<0.1%
TUJ1	49.5%	31.55
Nestin	83.25%	52.7%

Although both early (P4) and later (P40) passaged MSCs expressed neuronal markers, the P4 MSCs expressed higher levels of the MSC marker, CD90, and neuronal markers (TUJ1 and Nestin), while P40 MSCs had a higher expression of the MSC marker, CD 105.

**Table 2:** Flow Cytometry analysis following the DDI and IDI.

Marker	P4 Control	P4 DDI	P4 IDI	P40 Control	P40 DDI	P40 IDI
DAT	4.7%	45.3%	75.7%	9.4%	78.7%	50.9%
TH	16.9%	32.1%	19.1%	10.2%	35.8%	17.8%
MAP2	0.9%	15.8%	66.0%	1.4%	13.1%	9.1%
Nestin	80.4%	39.4%	75.4%	25.2%	76.3%	69.1%
lmx1a	60.8%	57.2%	78.4%	2.7%	64.7%	71.6%

Both the DDI and IDI increased the percentage of cells expressing dopaminergic markers, regardless of passage number.

**Table 3:** Flow Cytometry results following stage one.

Marker	P4	P40
DCX	99.2%	99.4%
Nestin	91.6%	84.8%
TUJ1	97.5%	97.3%

A high percentage of cells from early (P4) and later (P40) passaged expressed neuronal markers.

## Abbreviations

ANOVA	Analysis of Variance
BDNF	Brain derived neurotrophic factor
bFGF	Basic fibroblast growth factor
BHA	Butylated hydroxyanisole
BSA	Bovine serum albumin
CA	Catecholamine
cDNA	Complementary deoxyribonucleic acid
CMU	Central Michigan University
DA	Dopaminergic
DAT	Dopamine transporter
dcAMP	Dibutyryl cyclic AMP
DCX	Doublecortin
DDI	Direct Dopaminergic Induction
DMSO	Dimethyl sulfoxide
EGF	Epidermal growth factor
ELISA	Enzyme-linked immunosorbent assay
FACS	Flow cytometry
FBS	Fetal bovine serum
FCS	Forward scatter
FGF-8	Fibroblast growth factor-8
FITC	Fluorescein isothiocyanate
FNI	Field Neurosciences Institute
GAPDH	G-protein-regulated inward-rectifier potassium channel 2
GDNF	Glial-derived neurotrophic factor
GID	Graft induced dyskinesia
GIRK2	G-protein-regulated inward-rectifier potassium channel 2
HBSS	Hanks balanced salt solution
HD	Huntington’s disease
HS	Horse serum
hUMSCs	Human umbilical cord mesenchymal stem cells
IACUC	Institutional Animal Care and use Committee
IBMX	Isobutylmethylxanthine
ICC	Immunocytochemistry
IDI	Indirect Dopaminergic Induction
iPSCs	Induced pluripotent stem cells
K^+^	Potassium
L-DOPA	l-3,2-dihydroxyphenylalanine
LMX1a	LIM homeobox transcription factor 1
MAP2	Microtubule associated protein 2
MEA	Mutli-electrode array
MCH II	Major histocompatibility complex class II
FNI	Field Neurosciences Institute
GAPDH	G-protein-regulated inward-rectifier potassium channel 2
GDNF	Glial-derived neurotrophic factor
GID	Graft induced dyskinesia
GIRK2	G-protein-regulated inward-rectifier potassium channel 2
HBSS	Hanks balanced salt solution
HD	Huntington’s disease
HS	Horse serum
hUMSCs	Human umbilical cord mesenchymal stem cells
IACUC	Institutional Animal Care and use Committee
IBMX	Isobutylmethylxanthine
ICC	Immunocytochemistry
IDI	Indirect Dopaminergic Induction
iPSCs	Induced pluripotent stem cells
K^+^	Potassium
l-DOPA	l-3,2-dihydroxyphenylalanine
LMX1a	LIM homeobox transcription factor 1
MAP2	Microtubule associated protein 2
MEA	Mutli-electrode array
MCH II	Major histocompatibility complex class II
MSCs	Mesenchymal stem cells
NCID	Notch intracellular domain
NeuN	Neuronal nuclei
NSC	Neural stem cell
Nurr1	Nuclear receptor related-1 protein
PBS	Phosphate buffered saline
PD	Parkinson’s disease
PDGF	Platelet-derived growth factor
PEI	Polyethyleneimine
Pitx3	Pituitary homeobox 3
qRT-PCR	Quantitative reverse transcriptase polymerase chain reaction
RNA	Ribonucleic acid
SEM	Standard error
SHH	Sonic hedgehog
SCC	Side scatter
TH	Tyrosine hydroxylase
TTX	Tetrodotoxin
TUJ1	B-tubulin III
VTA	Ventral tegmental area
αMEM	Minimum essential medium alpha modification
